# Functionally Relevant Differences in Plasma Fatty Acid Composition and Expression of Cytotoxic and Inhibitory NK Cell Receptors between Healthy Young and Healthy Elder Adults

**DOI:** 10.3390/nu12123641

**Published:** 2020-11-26

**Authors:** Juan Bautista De Sanctis, Daciana Catalina Dumut, Danuta Radzioch, Marián Hajdúch

**Affiliations:** 1 Institute of Molecular and Translational Medicine, Faculty of Medicine and Dentistry, Palacky University, Hněvotínská 1333/5, 779 00 Olomouc, Czech Republic; marian.hajduch@upol.cz; 2 Department of Medicine, Division of Experimental Medicine, McGill University, 1001 DeCarie Boulevard EM 23242, Montreal, QC H4A 3J1, Canada; daciana.dumut@mail.mcgill.ca (D.C.D.); danuta.radzioch@mcgill.ca (D.R.); 3 The Research Institute of the McGill University Health Centre, Infectious Diseases and Immunity in the Global Health Program, 1001 DeCarie Boulevard EM 23242, Montreal, QC H4A 3J1, Canada; 4 Department of Genetics, McGill University, 1001 DeCarie Boulevard EM 23242, Montreal, QC H4A 3J1, Canada

**Keywords:** natural killer cells, ageing, saturated fatty acids, unsaturated fatty acids, killing activating receptors, killing inhibitory receptors (KIR)

## Abstract

(1) Background: In the healthy ageing, NK cell number is not modified; however, their spontaneous cytotoxicity decreases. We postulated that the age-dependent decline in metabolic activities might be responsible for this effect. (2) Methods: The fatty acid profile of 30 healthy young males (23 ± 4 years old, BMI 22.1 ± 1.3) and 30 older males (63 ± 5 years old, BMI 22.9 ± 2.5) donors were evaluated along with the expression of killing (KR) and inhibitory NK receptors (KIR) at basal level and after cultivation with fatty acids for 24 h. (3) Results: Significantly higher levels of oleic (*p* < 0.01), arachidonic (*p* < 0.001), lignoceric (*p* < 0.001), and nervonic acids (*p* < 0.0001) and significantly lower levels of docosapentaenoic and docosahexaenoic acids (*p* < 0.01) were found in elders as compared to young adults. At basal levels, significant (*p* < 0.005) differences in KR and KIR expression were encountered; 12/16 antigens. Treatment of cells with saturated fatty acids or arachidonic acid (AA) significantly enhanced KR expressions (*p* < 0.001). AA treatment decreased inhibitory KIR expression while docosahexaenoic, and eicosapentaenoic acid increased them. (4) Conclusions: Changes in fatty acids blood levels, and KR and KIR expression in NK cell, are age-dependent. Supplementation of NK cells with eicosapentaenoic or docosahexaenoic acid enhanced inhibitory KIR receptors’ expression which may improve their cell function.

## 1. Introduction

NK cells are a subpopulation of lymphocytes different from T cells that are involved in innate and adaptive immune responses (1). Their main characteristic, aside from the lack of CD3, is the expression of NCAM (CD56) and FcγRIII (CD16). These cells are involved in immune surveillance of cancer and infectious diseases, tolerogenic pregnancy, and several acute and chronic diseases [1,2,3]. Different subpopulations, based upon CD56 and CD16 expression, have been described [1,2,3]. These subpopulations seem to be modulated depending on antigens, cytokines and tissue milieu [1,2,3].

In healthy aged adults, the number of NK cells has been reported to be slightly increased as compared to young adults; however, NK cell cytotoxic responses were shown to be decreased [4,5]. This difference could be due to either a predominant subtype of CD56 NK cells or a decrease in the activation of signalling pathways responsible for spontaneous cytotoxicity [1,2,3,4,5]. No clear evidence of NK deficiency has been reported in healthy ageing subjects [6].

Some authors have postulated the use of the term ‘inflammaging’ to describe changes in the ageing immune response [7,8]. The hallmark of inflammaging is a pro-inflammatory metabolism. Changes in arachidonic acid and saturated fatty acids have been related to subtle but chronic inflammation (metabolic syndrome) and ageing [7,8,9,10]. Cardiovascular disease, hypertension, and atherosclerosis are a consequence of inflammaging [7,8,9,10]. Changes in lipid and lipoprotein metabolism affect all leukocytes, including NK cells [10,11,12].

In general, the lipid profiles of young adults and healthy elderly adults are similar, at the levels of triglycerides, total cholesterol, LDL and HDL cholesterol. However, there are significant differences observed at the level of circulating fatty acids in blood plasma in the different lipid moieties [13,14,15]. Changes in fatty acid composition in plasma suggest a dissimilar metabolic uptake and/or catabolism/anabolism [13,14,15]. These changes influence immune responses [16,17,18,19].

Our group has shown that NK cells express lipoprotein lipase (LPL), a key enzyme in triglyceride degradation [20]. Hence the uptake and degradation of lipoproteins and the uptake of fatty acids are essential for NK cell metabolism and function [12,20]. One plausible hypothesis that has been proposed is that the content of arachidonic acid on lipoproteins increases with age predisposing inflammatory conditions in the elders [16,17,18,19]. Decreases in ω3/ω6 fatty acid ratios could be responsible for inflammaging and the impaired response of NK cells [16,17,18,19].

Several authors have studied NK cell cytotoxic responses and the role of/ inhibitory receptors in ageing to explain differences in cell function [21,22,23,24,25,26,27,28,29]. The primary receptor is CD94 which associates with one of the seven members of NKG2. The complex CD94NKG2 can generate either activation or inhibition [21]. The main complexes encountered are CD94NKG2A (inhibitory), and CD94NKC or NKG2D (activatory) [21]. The inhibitory complex binds to HLA-E molecules while the activatory complexes bind to different ligands expressed in the target cells inducing NK cell activation. A mild but significant decrease in the expression or/and function of NKG2D has been reported among elders; consequently, the possible decline in spontaneous cytotoxicity [25,26,27,28].

Several HLA, and non-HLA specific activator and inhibitory receptors are expressed on NK cell surface [21,22,23,24,25,26,27,28,29]. Killer immunoglobulin-like receptors (KIR) are HLA-specific receptors involved in cytotoxic and tolerogenic responses of T and NK cells. KIR receptors have been involved in several physiological and pathological responses; however, their expression and function in ageing are controversial [21,23,24,25,29].

Within the non HLA receptors, NKp receptors are involved in NK cell recognition and killing of target cells. Consequently, their increase in expression has been related to the effectiveness of tumour killing [21,22,23,24,25]. NKp44 is expressed in a low percentage on NK cells and is generally upregulated in activated cells [21,22,23,24,25]. A significantly decreased expression of the activating receptor NKp30 and NKp46 was reported in NK cells from elderly individuals as compared to young adults. The decrease may also contribute to the reduced cytotoxicity recorded in the elderly [21,22,23,24,25,26,27,28]. However, this hypothesis has not been fully confirmed.

Other important non-HLA receptors involved in NK cell functions are CD160 and CD161. CD160 is a glycoprotein expressed in the NK cell population CD56 dim CD16+ bright, and it is responsible for cell activation and pro-inflammatory cytokine secretion [21,29,30]. The receptor CD161 is related to cell activation, mainly through PKC, and it has been described as an inhibitory receptor [31]. Expression of CD161 receptor is increased in ageing [26]. The function of this receptor is still controversial since it increases upon cell activation, and yet it has been described as an inhibitory receptor [26]. There is minimal information in the literature on the prevalence of higher or lower expression levels of other NK receptors in ageing and the possible effect of lipid metabolism.

The expression of two adhesion receptors CD62L (selectin) and CD11b (integrin) were reported to be slightly decreased with ageing suggesting a diminished NK cell tolerogenic function [21,22,23,24,25,26,27]. The expression of both receptors has been related to CD56 dim cell subpopulation [26].

Fatty acids induce protein kinase C activation (PKC) and NFkB modulation [32,33,34]. CD69, an antigen expressed upon leukocyte cell activation, is a useful marker of PKC induced activation. CD69 is similarly expressed in NK cells from healthy young and older adults suggesting that the PKC pathway is not affected by ageing [25]. Omega 3 fatty acids have been shown to downregulate NFkB activation reducing the transcription and secretion of inflammatory cytokines [34].

The present study aimed to investigate the differences between the most relevant fatty acids in healthy ageing, comparing healthy elders to healthy young adults of the same gender not involving possible hormonal effect. The second objective is to assess the differences in NK cell antigen expression between those groups using purified cells. The third objective is to ascertain the impact of those specific fatty acids on the positiveness of different receptors on purified NK cells from both groups of donors. We hypothesise that the changes in circulating fatty acids in ageing affect antigen expression of NK cells and consequently NK functions.

## 2. Materials and Methods

### 2.1. Reagents

Bovine albumin fatty acid-free and the fatty acids (1) palmitic acid (C16:0), (2) stearic acid (C18:0), (3) oleic acid (C18:1), (4) arachidic acid (20:0), (5) arachidonic acid (20:4), (6) eicosapentaenoic acid (20:5), (7) dosapentanoic acid (C22:5). (8) docosahexaenoic acid (C22:6), (9) lignoceric acid (C24:0) nervonic acid (C24:1), and (10) heptadecanoic acid (C17:0), served as an internal standard, and were purchased from Sigma Aldrich.

The following antibodies were acquired from Beckman Coulter CD3-CD16/CD56+, anti- CD158a anti-KIR2DL1/S1 (clone EB6B and clone Z27.3.7), anti-CD94 (clone HP-3B1) and anti NKG2A (CD159a, clone Z199). From Becton Dickinson, were purchased following antibodies: anti-KIR3DL1 (clone DX9), anti NKp30 (clone P30-15), anti NKp46 (clone 9E2), anti CD160 (clone BY55). Anti-human anti-CD158b (anti-KIR2DL3, clone DX27), CD 158f (anti-KI2DL5, clone UP-R1), anti-NKP44 (clone P44-8), and anti CD161 (HP-3G10) were purchased from Biolegend. The anti NKG2D (Clone # 149810) was purchased from R&D Systems. The antibodies anti-CD158e1/e2 KIR3DL1 (clone REA168), anti-KIR2DS4/CD158f (clone JJC11.6) were purchased from Miltenyi Biotech. The anti-mouse Fab FITC, PE, PE-Cy7 and the isotype controls mouse IgG1, IgG2 labelled with FITC, PE or PC5 were purchased from Becton Dickinson.

### 2.2. Blood Samples

Blood samples were obtained from 60 healthy male human normolipemic donors (body mass index 22.1 ± 1.3); 30 from the young adult group (23 ± 4 years old), and 30 from the healthy elders’ group (63 ± 5 years old). The volunteers were members of a marathon club and were continuously screened for chronic and infectious diseases. Pulmonary function, assessed by spirometry, and electrocardiograms were normal. Laboratory tests were within the normal range. All volunteers signed the written consent, and the local Ethical Committee approved the study (code HS101022017).

The number of lymphocytes in the blood was significantly (*p* = 0.02) lower in the elders as compared to the young adult donor group (Appendix A). The percentages of NK cells were similar between the two groups, 10.1 ± 1.0% for cells derived from young adult donor group vs. 9.6 ± 1.3% from elders. However, the amount of NK cells per mm^3^ was significantly (*p* = 0.001) lower in the elders (234.0 ± 36.0) as compared to the young adult donor group (268.1 ± 39.2), Table S1, Appendix A.

The volunteers were on a strict diet designed by the nutritionist according to the guidelines of the American Dietetic Association; Dietitians of Canada for marathon runners [35].

### 2.3. Fatty Acid Analysis in Plasma

Non-esterified fatty acids in plasma were analysed using the standard fatty acid kit (Sigma-Aldrich) following manufacture’s instruction. The concentrations of non esterified fatty acids were similar in both studied groups (Table 1).

The total fatty acid content was assessed using gas chromatography after methylating them. The samples (0.1 mL) were mixed with 1.9 mL of chloroform/methanol (2:1 *v*/*v*) and 1 mL of cold water, as described before [36]. Lipids were transferred to sterile glass tubes; chloroform was evaporated under nitrogen. Then, 2 mL of methanolic 5% hydrochloric acid was added. After shaking, the mixture was incubated for two h at 100 °C. After cooling to room temperature, the methyl derivatives were extracted twice with 2 mL of n-hexane, dried under nitrogen, and finally dissolved for analysis. Methylated samples were subjected to a GC/MS system in a quadrupole-orbitrap mass spectrometer (Thermo Scientific). The GC was a Hewlett Packard with a WCOT capillary column, Supelco-10, 35 m × 0.5 mm, 1 μm film. The samples were processed at an initial temperature of 100 °C, and subsequently, processing temperature was increased to 240 °C. The samples, 1 µl, were injected to the GS/MS. Mass spectra identification was set in the selected ion monitoring mode. The standard curve for each fatty acid was performed, and C17:0 served as an internal standard.

### 2.4. NK Cells

NK cells were purified using RosetteStep™ purification kit (Stem Cell Technologies, Vancouver, BC, Canada) using one-step Ficoll-hypaque. After separation, the cells were washed with PBS and counted. The obtained cells were similar in both groups, 94 ± 2.2% CD3^−^CD56^+^CD16^+^. The NK cells were not further separated to subpopulations of CD56 (dim or bright) since treatment has been suggested to modify the expression of this antigen.

### 2.5. Incubation of NK Cells with the Fatty Acids

NK cells were treated for 1 h with 5% bovine albumin, fatty acid-free, to set the cells in basal condition. Then, the cells were washed and adjusted to 2 million/mL and incubated for 18 h in RPMI 1640 media containing 10% bovine fatty acid-free albumin previously mixed with the fatty acid. The final concentration was 10 μM of fatty acid in the tube; this concentration was found to be optimal, Supplementary Table S3 and reference [12]. The fatty acids used were (1) palmitic acid, PA (C16:0), (2) stearic acid, SA (C18:0), (3) oleic acid, OA (C18:1), (4) arachidic acid, ARA (20:0), (5) arachidonic acid, AA (20:4), (6) eicosapentaenoic acid, EPA (20:5), (7) docosahexaenoic acid, DHA (C22:6), (8) lignoceric acid, LA (C24:0) and (9) nervonic acid, NA (C24:1).

The cells isolated from the blood of donors were washed and adjusted to 2 million/mL. Then, 0.1 mL were added to flow cytometry tubes, to analyse expression level and frequency of expression of different markers, including CD94, NKG2A, NKG2D, NKp30, NKp44, NKp46, CD160, CD161, CD62L, CD69, CD11b and the Killing Inhibitory Receptors (KIR) KIR2/DL1S1 (CD158a), KIR2/DL3 (CD158b), KIR3DL1 (CD158e1/e2), KIR2DL5 (CD158f) and KIR2DS4 (CD158i). The incubation was done in the dark at 4 °C. The cells were then washed twice before the analysis. The primary labelled antibodies used were either FITC, PE or PC5, and maximum three-colour analysis was performed in each tube. The incubation of cells with antibodies was accomplished using the standard technique (using the concentration and protocol recommended by the manufacturer), and the cells were analysed by flow cytometry using XL Beckman Coulter instrument.

### 2.6. Statistical Analysis

For the assessments of fatty acids and basal expression of receptors, the analysis two-tailed Student’s *t*-test (young adults vs. elders) was used. For the analysis of fatty acid treatment, one way ANOVA was used. A Bonferroni post-test analysis was used to compare the effect of treatment in each group, treatment with BSA with no fatty acids was used as a control for each analysed sample. The analysis was performed using Graph Pad Prism (version 5.0; Graph Pad Software, San Diego, CA, USA). The *p*-values less than 0.05 were considered significant.

## 3. Results

### 3.1. Circulating Fatty Acids

The amount of non-esterified fatty acid did not differ between groups (Table 1). However, in healthy elders, a substantial increase in plasma content (esterified) long-chain fatty acids was observed, lignoceric, nervonic, arachidonic, and oleic acids. Moreover, a significant decrease in docosapentaenoic and docosahexaenoic acid levels was recorded as compared to healthy young adults. These differences were independent of the general plasma lipid profile analysis (lipoproteins, cholesterol) of the volunteers; no dyslipidemias were encountered.

### 3.2. NK Cell Number and Purification

The total amounts of NK cells (CD16^+^C56^+^/CD3^−^) present in peripheral blood of the two groups were 8–12%; however, the total amount of NK cells was lower in the blood of the elder group compared to the young adult group (Appendix A). In both groups, cell purity was 94 ± 2% (Appendix A). In Appendix A, Figure S1 illustrates the results of purification of NK cells using blood from young adult donors. Figure S2 represents the percentage of cells expressing different antigens expressed in NK cells of young adults and elders.

### 3.3. Basal Expression of Killing and KIR Receptors

There were significant changes in the expression, in both percentage and mean channel fluorescence intensity (MFI) of several antigens and receptors, as shown in Table 2, which illustrates the percentages of cells expressing each antigen at the basal conditions. Significance is recorded in both parameters. Only four markers did not significantly differ in their expression between elders and young adult groups: NKp30, NKp46, CD161, and KIR2DL3 (CD158b). Among these four markers NKp30, and 46, are activating, CD161 is inhibitory, and KIR2DL3 is a killing inhibitory receptor.

Supplementary Figure S2 illustrates the expression of the different killing activating and inhibitory receptors on the cells harvested from young adults and elders. In general, antigens related to cell activation (NKp 44, CD160, CD62L, CD69) were found to be expressed at a higher level on the cells from the elders compared to the young adult group. The surface expression levels of CD94 and NKG2D, as well as of CD158e1/e2, CD158i, and of CD11b were significantly lower in the cells harvested from elders versus young adults group. These decreased percentages in the elders coincided with the higher level of expression of inhibitory receptors NKG2A, CD158a and CD158f.

**Figure 1:**
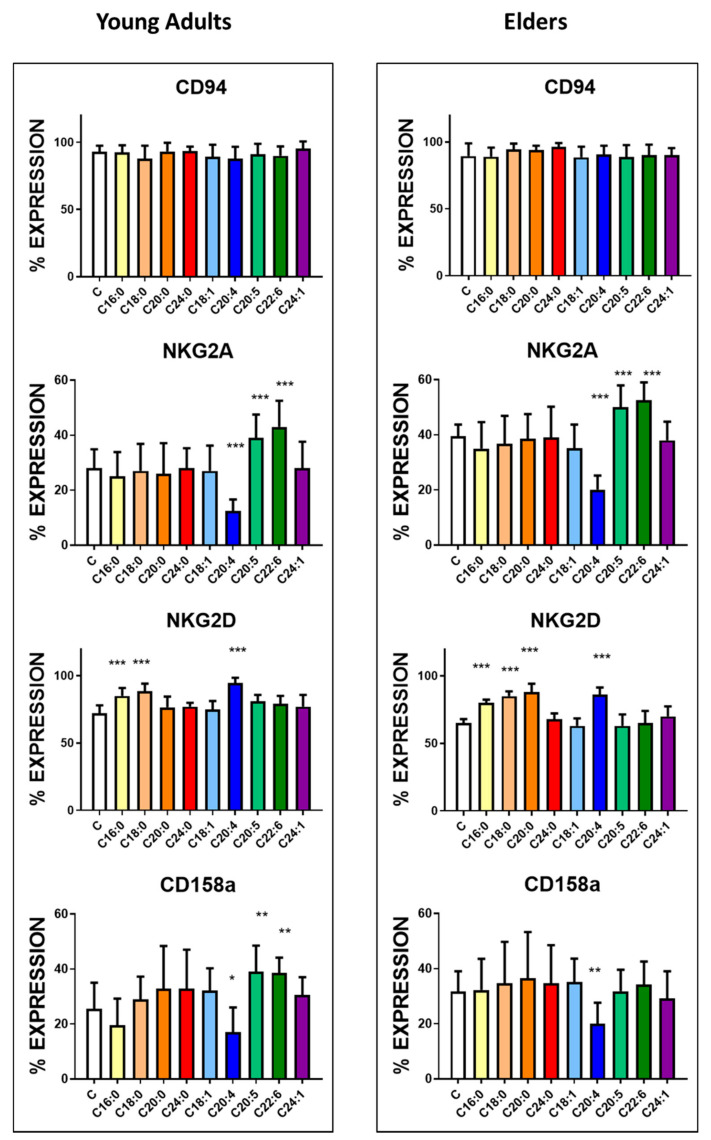
Effect of fatty acids treatment on the mean percentage expression of CD94, NKG2A, NKG2D and CD158a antigens. NK cells purified from the blood of the healthy young adults (23 ± 4 years old) and healthy elders (63 ± 5 years old) were treated with 10 µg/mL concentration of the following fatty acids for 24 h. The expression of the receptors was assessed by flow cytometry. The results represent the percentage mean and SD for each group (*n* = 30). The treatments were compared by one way ANOVA. Bonferroni significance post-test are represented. * *p* < 0.01, ** *p* < 0.001 and *** *p* < 0.0001. No significant differences are recorded for CD94. The mean channel fluorescent intensity (MFI) is represented in Supplementary Figure S3.

In general, fatty acid treatment did not modify the expression of CD94 in the two groups, as illustrated in Figure 1, Supplementary Figure S3. However, supplementation with fatty acids led to modifications of the percentage of positive cells expressing NKG2A and NKG2D receptors among NK cells in both groups. As shown in Figure 1 and Supplementary Figure S3, treatment with AA decreased the expression of NKG2A while increasing expression of NKG2D receptors. On the contrary, treatment with either DHA or EPA increased the expression of NKG2A without affecting the percentage of cells with positive NKG2D receptor expression. In both groups, all saturated fatty acids treatment tested increased NKG2D expression.

**Figure 2:**
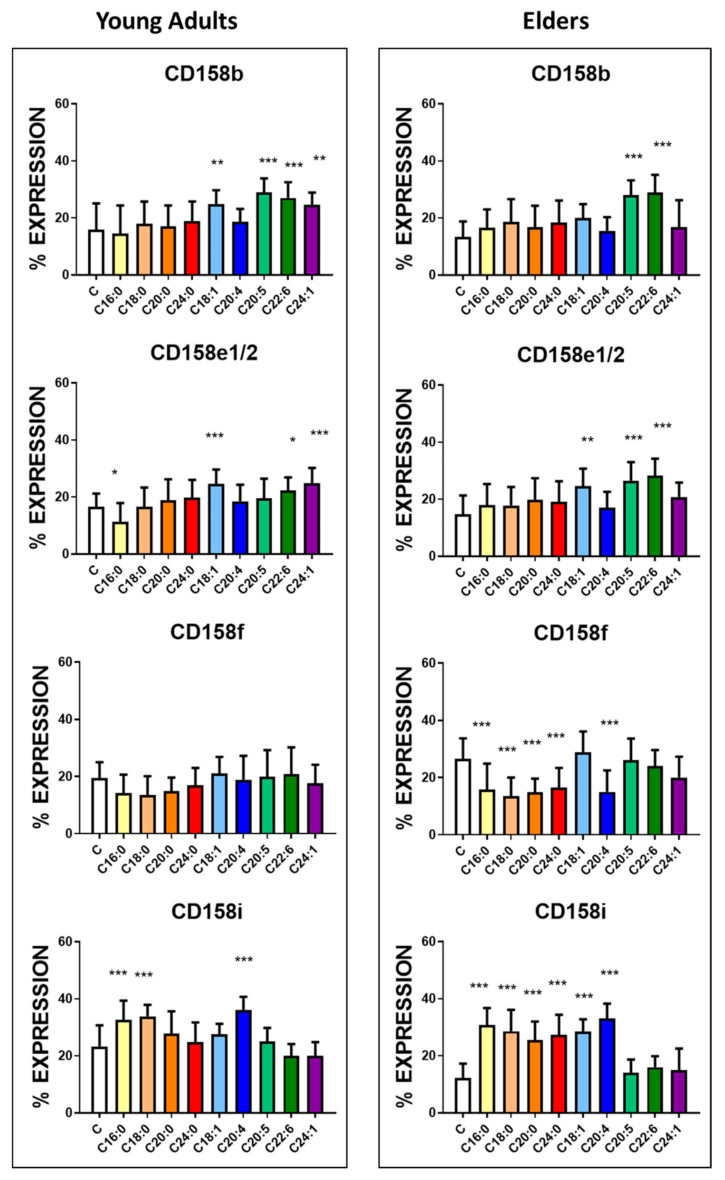
Effects of fatty acids on the expression of CD158 antigens in NK cells. The receptors are inhibitory except for CD158i. NK cells purified from the blood of the healthy young adults (23 ± 4 years old) and healthy elders (63 ± 5 years old) were treated with 10 µg/mL concentration of the following fatty acids for 24 h. The expression of the receptors was assessed by flow cytometry. The results represent the percentage mean and SD for each group (*n* = 30). Treatments were compared by one way ANOVA. Bonferroni significance post-test are represented. * *p* < 0.01, ** *p* < 0.001 and *** *p* < 0.0001. The mean channel fluorescent intensity (MFI) is represented in Supplementary Figure S3.

For inhibitory receptors of the CD158 family (Figure 1 and Figure 2, Supplementary Figure S3), the induction and inhibition induced by treatment by fatty acids were not different from the effects observed on NKG receptor family member. CD158a expression (Figure 1, Supplementary Figure S3) was increased upon the treatment with EPA and DHA only in the cells harvested from healthy young adults. In contrast, no change in the percentage of positive cells was observed when the cells from the elders were treated. The treatment with AA significantly decreased the expression of this receptor in both groups.

In contrast, upon DHA and EPA incubation, the increase in the inhibitory receptor CD158b expression (Figure 2, Supplementary Figure S3) was observed in both groups. In the young adult group, treatment with monosaturated fatty acids, OA and NA, leads to an increase in the positiveness of CD158b on their surface.

Treatment of NK cells with OA and DHA increased the positiveness of the inhibitory receptor CD158e1/e2 in both groups (Figure 2, Supplementary Figure S3). The supplementation with EPA increased the expression of this receptor only in NK cells harvested from the blood of elders. In contrast, the treatment with NA induced a similar effect but only in cells harvested from the young adult group. Treatment with PA decreased the expression CD158e1/e2 only in the of NK cells harvested from the blood of young adults. The other inhibitory receptor CD158f was significantly downregulated in the NK cells harvested from elders in the presence of all saturated fatty acids and AA (Figure 2, Supplementary Figure S3).

Regarding the activating receptor CD158i, PA, SA, and AA significantly increased the expression of this receptor in both groups; however, ARA, LA and OA significantly increased the positiveness of this receptor only in the NK cells harvested from the elder group (Figure 2, Supplementary Figure S3).

**Figure 3:**
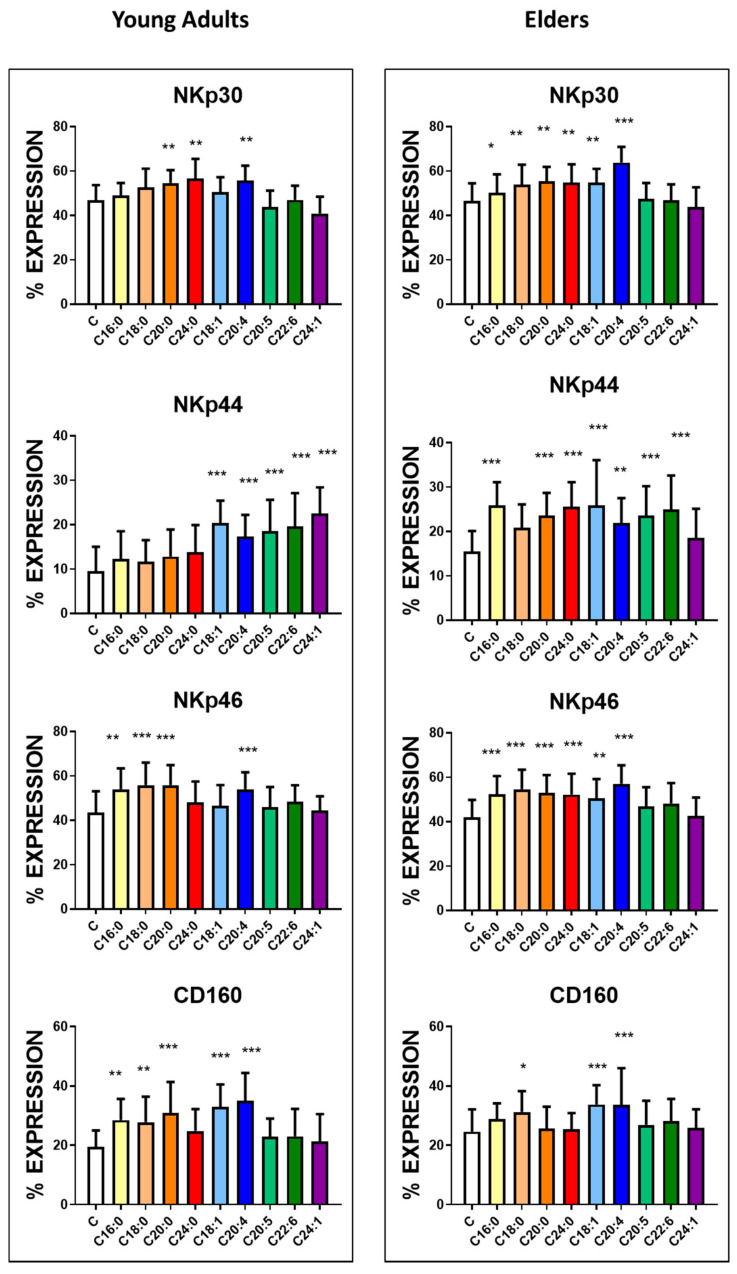
Expression of NKp30, 44 and 46 and CD160 upon treatment with fatty acids and assessed by flow cytometry. NK cells purified from the blood of the healthy young adults (23 ± 4 years old) and healthy elder (63 ± 5 years old) volunteers were treated with 10 µg/mL concentration of the following fatty acids for 24 h. The expression of the receptors was assessed by flow cytometry. The results represent the percentage mean and SD for each group (*n* = 30). The treatments were compared by one way ANOVA. Bonferroni significance post-test are represented. * *p* < 0.01, ** *p* < 0.001 and *** *p* < 0.0001. The mean channel fluorescent intensity (MFI) is represented in Supplementary Figure S4.

Analysis of the expression of cytotoxic receptors that belong to the NKp family (Figure 3, Supplementary Figure S4) revealed that the treatment with AA significantly increased the positiveness of all the receptors on the NK cells independently of the donor group. Except for SA in the case of NKp44 receptor expression, the saturated fatty acids treatment enhanced the appearance of these receptors in the cells derived from the elders. In NK cells from young adults, NKp 44 expression was enhanced by mono and polyunsaturated fatty acids. Specifically, the treatment with ARA increased the expression of NKp30 and NKp46; treatment with LA increased expression of NKp30, and supplementation with PA and SA increased expression of NKp46.

**Figure 4:**
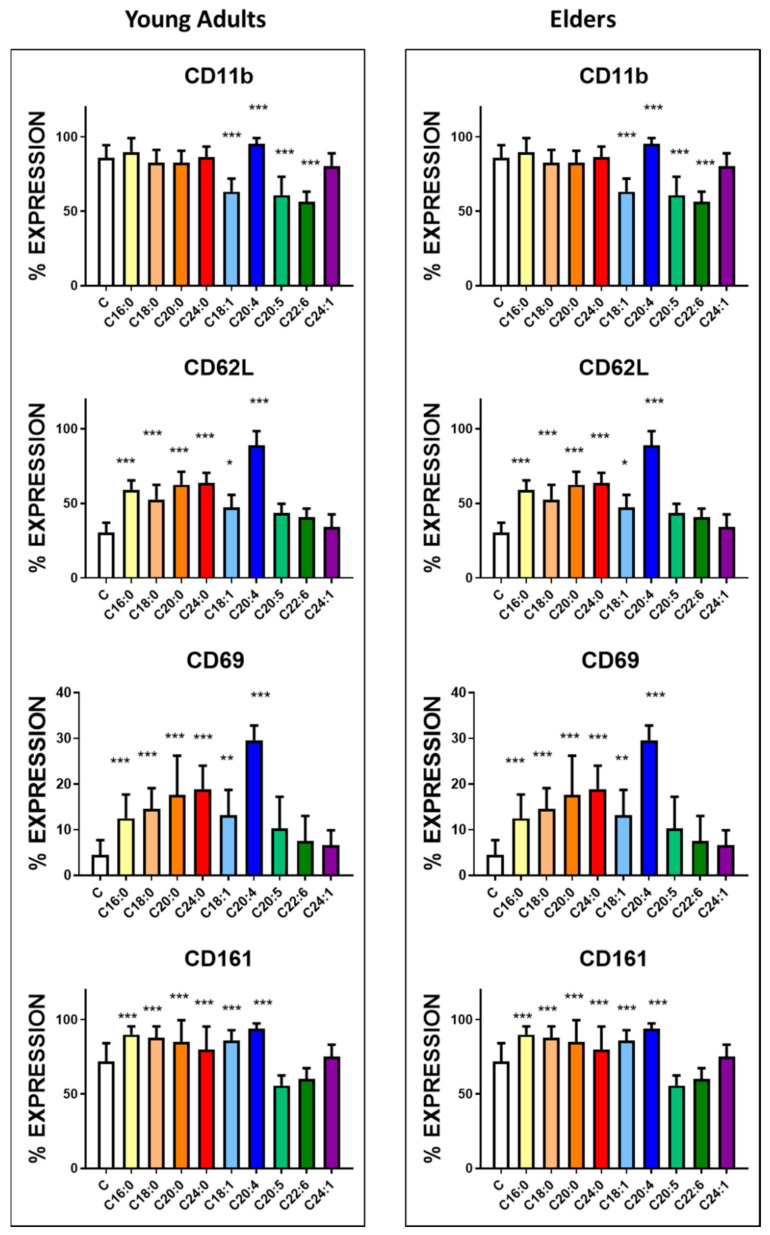
Expression of CD11b, CD62L, CD69 and CD161, markers of cells activation, upon treatment of NK cells with fatty acids. NK cells purified from the blood of the healthy young (23 ± 4 years old) and healthy elder (63 ± 5 years old) volunteers were treated with 10 µg/mL concentration of the following fatty acids for 24 h. The expression of the receptors was assessed by flow cytometry. The results represent the percentage mean and SD for each group (*n* = 30). The treatments were compared by one way ANOVA. Bonferroni significance post-test are represented. * *p* < 0.01, ** *p* < 0.001 and *** *p* < 0.0001. The mean channel fluorescent intensity (MFI) is represented in Supplementary Figure S4.

The expression of the CD160 receptor (Figure 3, Supplementary Figure S4) became enhanced in the presence of PA, OA, AA and SA and only in the cells harvested from the healthy young adult group. AA treatment also upregulated other antigens, including CD11b and CD62L, involved in NK cell differentiation and recruitment (Figure 4, Supplementary Figure S4). CD11b was also upregulated by SA, ARA, LA but only in the NK cells derived from the blood of the healthy elder group. CD11b downregulation was observed after treatment with OA, EPA and DHA in NK cells from the blood of both groups. Treatment with all tested saturated fatty acids and OA enhanced the expression of CD62L, and interestingly, no downregulation was recorded in the presence of EPA or DHA.

Two receptors expressed upon cell activation, CD69 and CD161, were upregulated in NK cells derived from both experimental groups when the cells were stimulated with saturated fatty acids, OA and AA (Figure 4, Supplementary Figure S4).

**Figure 5:**
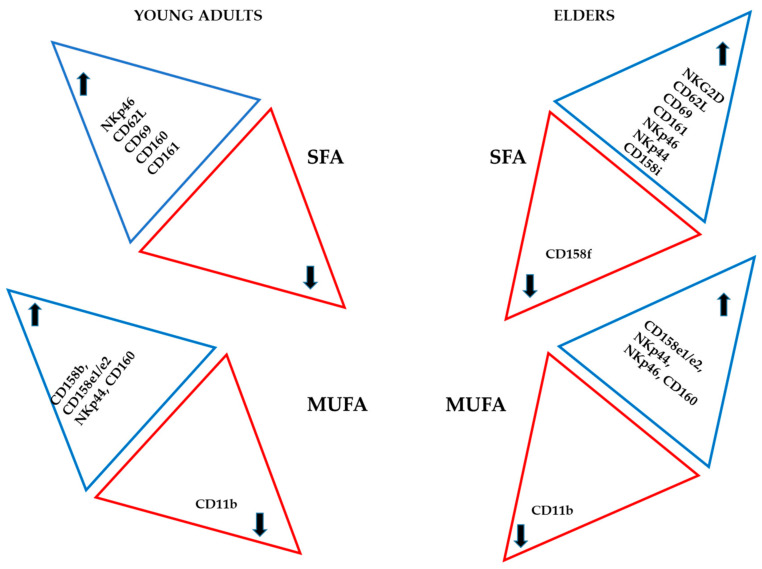
Effect of saturated (SFA) and w9 monosaturated fatty acids (MUFA) on the expression of different antigens in NK cells from healthy young adults and healthy elder subjects. The arrows indicate an increase or decrease of the antigens. SA corresponds to saturated fatty acids, w9 to OA and NA.

**Figure 6:**
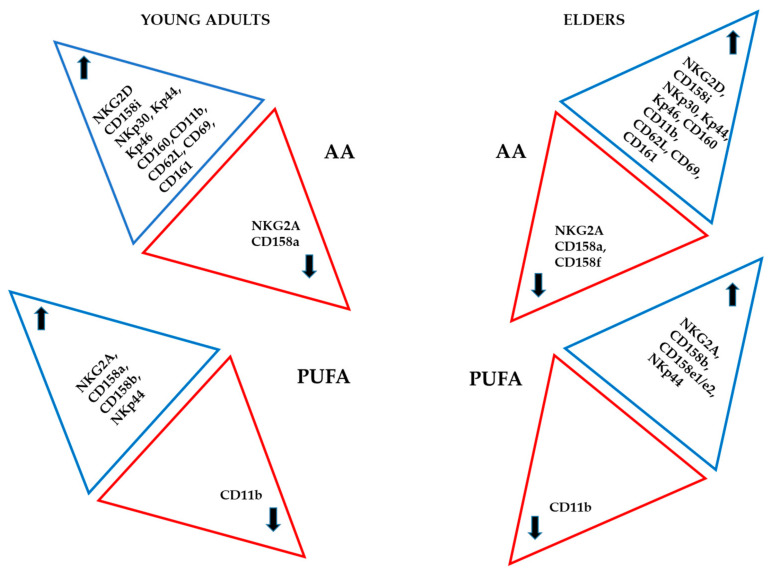
Schematic illustration of the effect of arachidonic acid (AA) and PUFA, polyunsaturated (PUFA) w3 fatty acids (DHA and EPA) on the expression of different antigens in NK cells from healthy young adults and healthy elder volunteers. The arrows indicate an increase or decrease of the antigens. AA corresponds to arachidonic acid, and w3 corresponds to DHA and EPA.

Figure 5 and Figure 6 and Supplementary Table S2 summarise the effect of the treatment of NK cells with different fatty acids on the expression of the various receptors assessed. In both groups, saturated fatty acids and AA induced the highest amount of antigens on NK cell membrane.

It is essential to mention that in the cells derived from the healthy elder subjects, the increase in the percentage of positive cells expressing killing receptors was significant. In the young adult group, the cell activation markers expression was enhanced. The effect of w9 fatty acids was evident in a group of killing receptors, and only a mild increase in one of the inhibitory receptors was observed in both populations. CD11b expression decreased following treatment with monosaturated fatty acids in the cells derived from both groups.

In general, the treatment with AA enhanced expression of most of the similar antigens in the NK cells from both experimental groups, with some exceptions. For example, the positiveness of NKG2A, CD158a, and CD158f were decreased following AA treatment only in the NK cells derived from healthy elder volunteers.

Finally, following treatment of NK cells with w3 fatty acids, an increase in the expression of NKG2A, CD158b and NKp44 was observed in the treated cells derived from the NK cells of both donor groups.

Treatment with w3 fatty acids increased expression of CD158a only in NK cells derived from healthy young adults, whereas CD158e1/e2 expression was enhanced following w3 fatty acids treatment only in the NK cells derived from the elders’ group. Treatment with w3 fatty acids downregulated CD11b, a marker for NK activation, in both groups.

The only marker which was downregulated upon two treatments in both experimental groups is CD11b. 

There was no correlation between the expression of activating receptors and inhibitory receptors between the two groups.

## 4. Discussion

Inflammaging has been defined as a pro-inflammatory status during ageing. It is characterised by an increase in cell activation markers’ expression, pro-inflammatory cytokine secretion, and a decrease in inhibitory receptors’ expression [7,37]. In ageing, there is a general statistically significant reduction in the number of circulating leukocytes and lymphocytes and an increase in neutrophils [7,37]. In the present report, we recorded a mild, but significant reduction in lymphocyte population in the healthy elder group (63 ± 5 years old) compared to the healthy young adults’ group (23 ± 4-years old), which affected the number of NK cells despite having a similar percentage of circulating cells. After cell purification, a comparative analysis of antigen expression at the basal level and after incubation could be performed without interference from other leukocytes. Several activating and inhibitory receptors and other antigens were assessed in NK cells. The expression markedly differed among the two groups studied. The observed differences in the positiveness of NK cell receptors between the cells derived from healthy young adults and healthy elder subjects seem to be associated with metabolic differences between the two groups. The lipidomic differences, including fatty acids, age and gender, have been described [15,19,38].

When the two groups are compared, CD94 NKG2D, CD158i, CD158e1/e2 and CD11b were expressed at a significantly lower level in the cells derived from healthy elderly donors, whereas NKG2A, NKP44, CD160, CD158a, CD158f, CD62L and CD69 proteins were expressed at a higher level than in the cells from young donors. These variations are complex and may represent different responses to metabolic stimulation. The changes in antigen expression are not due to a simple modulation of subpopulations of the CD56 or CD16 dim or bright NK cells; it is a more complex response. It is suggested that the modulation of receptors is dependent upon the metabolic conditions and the general immune status of the individuals. However, ageing, even in healthy subjects, can make a crucial difference.

NK cells’ proliferative and cytotoxic responses were previously shown to be modulated by lipoproteins [12]. The reaction encountered was thought to be dependent on lipoprotein internalisation [12]. However, fatty acids were not assessed and in the presence of a triglyceride degrading enzyme, lipoprotein lipase [20], fatty acids could be responsible for the effect in proliferative and cytotoxic responses. In recent years, research has focused on NK cells subpopulations and their response to various stimuli, but little efforts have been made on metabolism and cell responses.

NK cells are involved in immune surveillance and tolerogenic reactions [1,22]. Even though it is clear that tissue milieu is responsible for cytotoxic or tolerogenic responses, the modulation of receptor expression may affect the fate and the role of these cells. Thus, a pro-inflammatory condition, along with modified fatty acid ratios, would affect NK receptor expression, and response to stimuli. Several reports have suggested a connection between NK cell’s metabolic changes and subclinical inflammation [39,40,41,42,43,44,45].

The presence of long-chain and very-long-chain fatty acid in the plasma of healthy elderly population has been described previously; however, no primary mechanism for this increase has been revealed [13,14,15]. In the elderly, an increase in oleic acid levels has been related to cardiovascular diseases [37,38,39,40,41]. Likewise, arachidonic acid increase [13,14,37] in elder was suggested to play an essential role in immune ageing [7,19,34,37,38]. Several reports focused on the importance of diet and its effects on the immune response in the elderly [9,10,17,37,38,39,40,41,42,43,44,45]. In particular, Thies et al. [42] have addressed the impact of fatty acid supplementation on NK cells. The authors concluded that polyunsaturated fish oil decreased NK cell cytotoxic activity significantly. Eicosapentaenoic acid was thought to be responsible for these effects, as documented previously [43]. Recently, docosapentaenoic acid has been analysed for inflammation resolution, and it is decreased in the elders. The supplementation of NK cells with docosapentaenoic was performed in the present manuscript, and future studies should ascertain its role on NK receptor expression and function [44].

Most of the NK cells, independently of the type of donor they come from, did not show any modulation of membrane CD94 expression independently of the fatty acid used. As expected, CD94 protein has been stable but may associate with inhibitory NKG2A or activating receptor NKG2D. NKG2A is significantly down-regulated by AA, independently of the group analysed in our study. It is clear that milieu enriched in DHA and EPA favour the expression of NKG2A and saturated fatty acids, whereas enrichment with AA coincides with an increase in NKG2D expression. The proteins CD62L, CD11b, and CD69 are antigens expressed generally in activated cells. The cells from the healthy elder subjects have significantly higher expression of CD62L and CD69 in the basal state, but lower positiveness of CD11b as compared to healthy young adults group, suggesting that the cells may be primed in the inflammaging milieu or they are exhausted. The antigen CD161 differs in the basal state and is increased in both groups upon supplementation. This antigen could be a compensatory receptor for NK cell function [24,25]. NK cell priming does not necessarily involve enhanced cytotoxic response. In the elders’ group, NK cells respond to saturated fatty acid incubation by expressing killing receptors and activation markers, suggesting that the cells may be modulated depending on the metabolic activity and/or tissue milieu. It is possible that in the elders’ group, supplementation with fatty acids may revert the NK cell exhaustion phenomenon.

Upon incubation with saturated fatty acids and AA, the increase in the expression of killing receptors is more pronounced than the down-regulation of the inhibitory receptors. Recently, Poznaski and Ashkar [45] have addressed an important question concerning NK cell subpopulations depending on metabolic activity. The fact that donors of both age groups are marathon runners is exceptional since it rules out their relative health status, including underlying diseases that might have an impact on NK response reported in other studies [4,5]. The use of female volunteers is part of another study of the group since hormonal modulation modifies lipid moieties [15] and affects the expression of NK receptors [29].

The expression of CD160 is essential in NK cell function. It is markedly enhanced following treatment with w9 fatty acids and AA in both groups and the following incubation with saturated fatty acids in the young adult group. The expression of CD160 has been related to NK cell activation [46]. This enhancement in cell activation could be associated with the increasing development of atherosclerosis [47]. These events may be related to the report of Steffen et al. [39] that circulating levels of OA are related to cardiovascular events. The reported data suggested that OA levels are increased in the elderly population.

The marked decrease in CD11b positiveness, observed upon monounsaturated and w3 fatty acids treatment, suggests that this antigen is an essential marker of NK function and activity. NK subpopulations expressing this marker may be more pro-inflammatory. Fu and coworkers [48], described CD11b as one of the critical antigens along with CD56dim for the NK subpopulation to become more prone to be cytotoxic. Even though other antigens are essential, the expression of CD11b has been well documented to be related to tumour cell binding [48]. However, the downregulation of killing receptors is not the only mechanism associated with decreased cell activation and cytotoxic response.

**Figure 7:**
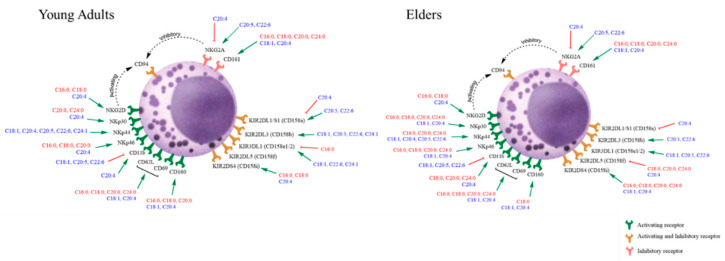
Graphical summary of the effect of fatty acid treatment on NK cell receptors in healthy young adults and elder subjects. The red, bar-headed, arrows indicate a decrease in the expression of receptors on NK cells. The green arrows indicate an increase in receptor expression. Receptors are classified as “activating receptors” in green, “activating and inhibitory receptors” in yellow and “inhibitory receptors” in red. Saturated fatty acids are shown in red. Unsaturated fatty acids are shown in blue.

Figure 7 illustrates the effect of the different fatty acids in healthy young adults and healthy elder subjects. The figure shows the possible receptor associations, induced by fatty acid supplementation, that may modulate NK function.

The results of different reports with ω3 fatty acids dietary intervention are difficult to analyse [19,38,39,40,41,42]. Most of the studies focus on the lipidomic profile and only assess part of the immune response and hence are difficult to compare. The expression of different cell receptors on NK cells has not been studied extensively in these conditions. One of the recent mechanisms postulated by Pinkosky and coworkers [49] is related to the activation of AMPK by long-chain fatty acids. Thus, metabolic changes in ageing can be responsible for the modulation of NK subpopulations; however, it cannot be ruled out that there are responses which are not related to ageing. Ageing may affect the timing or the impact of the reaction, but not the reaction itself.

Several authors [1,22,23,24,25,26,27,28], have described that NK cell response, seems to be hampered in aged individuals and patients with chronic diseases. The mechanisms involved depend on the disease, its stage, and therapy; therefore, analysing cell populations or antigen expression may provide only partial information on cell response and role in several conditions.

**Figure 8:**
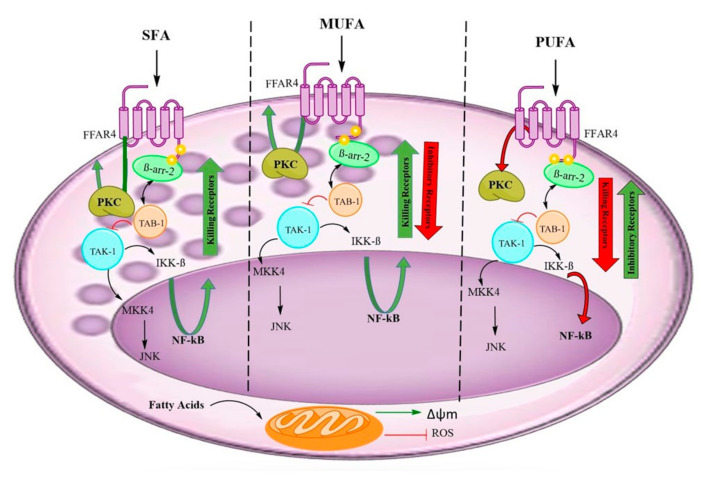
Graphical summary of the effects of fatty acids on NK cells. The postulated effects of saturated (SFA), monounsaturated fatty acids, w9 (MUFA) and polyunsaturated w3, fatty acids (PUFA). The effect of AA, not represented in the picture. FFAR4 corresponds to Free Fatty Acid Receptor 4, β arr-2 corresponds to β arrestin 2, PKC corresponds to Ptotein Kinase C, TAB-1 corresponds to Transforming growth factor beta-activated kinase 1(TAK)-binding protein 1, IIKB corresponds to Inhibitor Of Nuclear Factor Kappa B Kinase Subunit Beta, NFκB corresponds to Nuclear Factor Kappa B transcription factor, MKK4 corresponds to Mitogen-activated protein kinase (MAPK) kinase 4, JNK corresponds to c-Jun N-terminal kinase, Δψm corresponds to The mitochondrial membrane potential and ROS corresponds to Reactive Oxygen Species.

Figure 8 illustrates the general effect of 3 sets of fatty acids assessed. All the fatty acids generated PKC activation, assessed by CD69. However, the different fatty acids have differences in the activation or inhibition of NFkB. Arachidonic acid supplementation caused a particular effect on NK cells. AA induced an increase in killing receptors and diminished inhibitory receptors more than the other fatty acids, and probably it influenced different cellular pathways responsible for the recorded events. Future studies should unravel the signalling pathways responsible for the various effects observed.

It has been described that cytomegalovirus infection (CMV) modulates CD94, and KIR receptors in NK cells [50,51,52]. In the elderly volunteers that participated in the study, it cannot be ruled out that certain modifications in the receptor repertoire could be dependent upon latent CMV infection.

NK immunosurveillance seems to be dependent, at least in part, on cells’ metabolic condition. In the healthy elderly, due to inflammaging, the response against infections is not hampered; however, in different metabolic disorders, this response may be decreased. Tolerogenic NK cells may be more dependent on the milieu than the primary metabolic modulation. Several new hypotheses can be generated based on the results presented here. It would be fascinating to assess if dietary intervention would alter the modulation of NK cell responses to the benefit of elderly patients making them less vulnerable to infections and protecting them from the accelerated ageing process. Future studies are indeed required to test specific hypotheses in the context of ageing markers and immunity to specific viral infections.

## 5. Strengths and Limitations of the Study

The study represents the first effort to analyse the importance of different fatty acid encountered in the plasma of healthy elderly population with the expression of key receptors on NK cells in young and aged individuals. NK cells from healthy elders respond similarly to the young counterparts with few exceptions, and consequently, NK response could be induced by metabolic changes rather than impairment of cell function. The major limitation of the study is to assess possible NK cell populations which can be modulated upon fatty supplementation.

## 6. Conclusions

There are significant differences in the expression of cytotoxic and KIR receptors between the NK cells from healthy young adults compared to the healthy elderly group. These differences may be due to different metabolic responses since there were substantial differences in the blood fatty acid content in both groups. In general, saturated fatty acids and AA upregulate cytotoxic receptors and cell activation while EPA and DHA enhance expression of inhibitory receptors. CD11b is modulated by mono and polyunsaturated fatty acids suggesting a vital role of this antigen in NK responses.

## Figures and Tables

**Table 1:** Plasma fatty acids levels comparison between the two groups of healthy adults.

**Group of Individuals**	**Young Adults**	**Elders**	***p***
N	30	30	
Age	23 ± 4	63 ± 5	0.0001
BMI	22.1 ± 1.3	22.9 ± 2.5	0.95
Non sterified fatty acids µmol/L	398 ± 126	415 ± 125	0.5
**Total Fatty Acid (FAA)**	**FAA µmol/L**	**FAA µmol/L**	***p***
Palmitic (PA)	275.5 ± 15.6	250.1 ± 25.2	0.3
Stearic (SA)	29.2 ± 11.3	26.3 ± 16.1	0.5
Oleic (OA)	35.1 ± 14.4	50.4 ± 10.6	0.01
Arachidic (ARA)	6.1 ± 2.2	7.0 ± 4.6	0.8
Arachidonic (AA)	43.6 ± 18.2	68.3 ± 12.2	0.001
Eicosapentaenoic (EPA)	12.1 ± 5.4	9.4 ± 4.1	0.3
Docosapentaenoic (DPA)	21.8± 7.6	14.8± 6.1	0.01
Docosahexaenoic (DHA)	21.2 ± 5.1	10.2 ± 4.3	0.01
Lignoceric (LA)	5.1 ± 2.1	15.1 ± 7.4	0.001
Nervonic (NA)	11.3 ± 3.1	36.3 ± 4.1	0.0001

The table illustrates the characteristics of the groups and the number of fatty acids assessed in plasma of the volunteers. The statistical analysis was performed using a paired Student’s *t*-test.

**Table 2:** Expression of Killing Inhibitory Receptors (KIR) and activating receptors in NK cells derived from young adults and elders. The results represent the percentage mean and SD and Mean Channel Fluorescence Intensity (MFI) in linear units for each group (*n* = 30). The statistical analysis was accomplished using the paired Student’s *t*-test in both parameters analysed.

	Young Adults	Elders		Young Adults	Elders	
	%	%	*p*	MFI	MFI	*p*
CD94	95.5 ± 4.6	89.6 ± 9.5	<0.005	1250 ± 85	1075 ± 115	<0.0001
NKG2A	28.6 ± 3.9	39.5 ± 4.2	<0.0001	355 ± 55	501 ± 92	<0.0001
NKG2D	76.3 ± 6.1	65.1 ± 3.1	<0.0001	1072 ± 60	910 ± 55	<0.0001
NKp30	46.8 ± 6.9	43.4 ± 8.1	NS	1299 ± 69	1270 ± 81	NS
NKp44	9.5 ± 5.5	15.5 ± 4.6	<0.0001	430 ± 35	548 ± 46	<0.0001
NKp46	43.5 ± 9.6	41.9 ± 7.9	NS	1115 ± 56	1080 ± 79	NS
CD160	19.5 ± 5.5	24.6 ± 7.5	<0.005	495 ± 56	546 ± 65	<0.005
CD161	22.2 ± 12.2	25.2 ± 6.8	NS	1072 ± 82	1112 ± 68	NS
KIR2DL1/S1(CD158a)	25.5± 9.5	31.6 ± 7.4	<0.01	475 ± 58	572 ± 74	<0.0001
KIR2DL3 (CD158b)	15.8 ± 9.3	13.4 ± 5.4	NS	390 ± 33	376 ± 22	NS
KIR3DL1 (CD158e1/2)	16.6 ± 4.6	14.8 ± 6.5	<0.05	550 ± 46	504 ± 76	<0.01
KIR2DL5 (CD158f)	15.9 ± 5.5	26.5 ± 7.2	<0.0001	354 ± 45	388 ± 32	<0.005
KIR2DS4 (CD158i)	23.3 ± 7.4	12.2 ± 5.1	<0.0001	650 ± 74	390 ± 51	<0.0001
CD62L	30.5 ± 6.5	42.6 ± 7.2	<0.001	708 ± 95	805 ± 92	<0.0001
CD69	4.5 ± 3.2	8.9 ± 3.6	<0.0001	455 ± 75	583 ± 72	<0.0001
CD11b	85.9 ± 8.6	75.9 ± 9.5	<0.0001	2859 ± 96	2396 ± 125	<0.0001

## Supplementary Materials

The following are available online at https://www.mdpi.com/2072-6643/12/12/3641/s1, Figure S1: The figure illustrates a standard flow cytometry assessment after NK cell purification from a healthy young adult; 98% of the cells are NK cells, Figure S2: The figure represents the flow cytometry assessment of the different receptors in typical healthy young adults and a healthy elder individual, Figure S3: Effect of fatty acids treatment on the mean fluorescence intensity of CD94, NKG2A, NKG2D, CD158a, CD158b, CD158e1/2, CD158f, and CD158i antigens, Figure S4: Effect of fatty acids treatment on the mean fluorescence intensity of NKp30, NKp44, NKp46, CD160, CD11b, CD62L, CD69, and CD161 antigens, Table S1: Number of peripheral blood NK cells mm3 and the% purity after separation, Table S2: NK cell markers modulation by fatty acid treatment in cells isolated from the blood of healthy young adults and healthy elder subjects, Table S3: Effect of different concentrations of fatty acid on CD94 expression and cell viability.

## Appendix A

The number of samples required for the analysis was based on the following formula

$$\mathrm{Number}\;\mathrm{of}\;\mathrm{samples}\;\mathrm{needed}\;(n)=\frac{Z^{2}{}_{\alpha }\times p_{0}\times q_{0}}{D^{2}}$$

the value of Z^2^_α_ is 1.96 for the significance of 0.05, p_0_ is set to be 0.1 (according to the literature) q_0_ in 0.8 with a maximum tolerated difference D of 0.1.

Thus, $n=\frac{{\left(1.96\right)}^{2}\times 0.1\times 0.8}{{\left(0.1\right)}^{2}}=30.7$ the minimum required samples.
