# Supplementary material for: Functionally Relevant Differences in Plasma Fatty Acid Composition and Expression of Cytotoxic and Inhibitory NK Cell Receptors between Healthy Young and Healthy Elder Adults
Source: Nutrients. 2020 Nov 26;12(12):3641. doi: 10.3390/nu12123641 (PMC7759996; doi:10.3390/nu12123641)
Supplement: Supplementary file 1 [file nutrients-12-03641-s001.pdf]

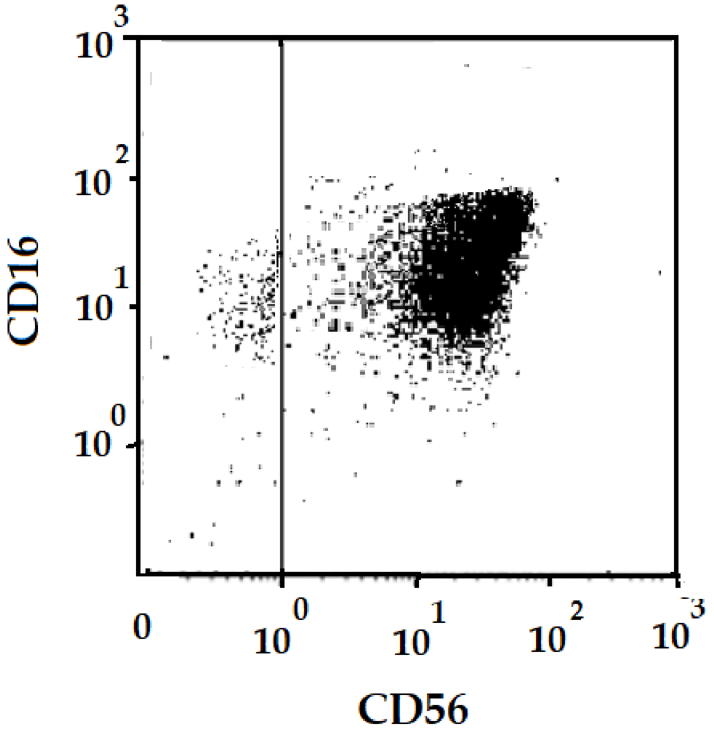

Figure S1. The figure illustrates a standard flow cytometry assessment after NK cell purification from a healthy young adult; 98% of the cells are NK cells.

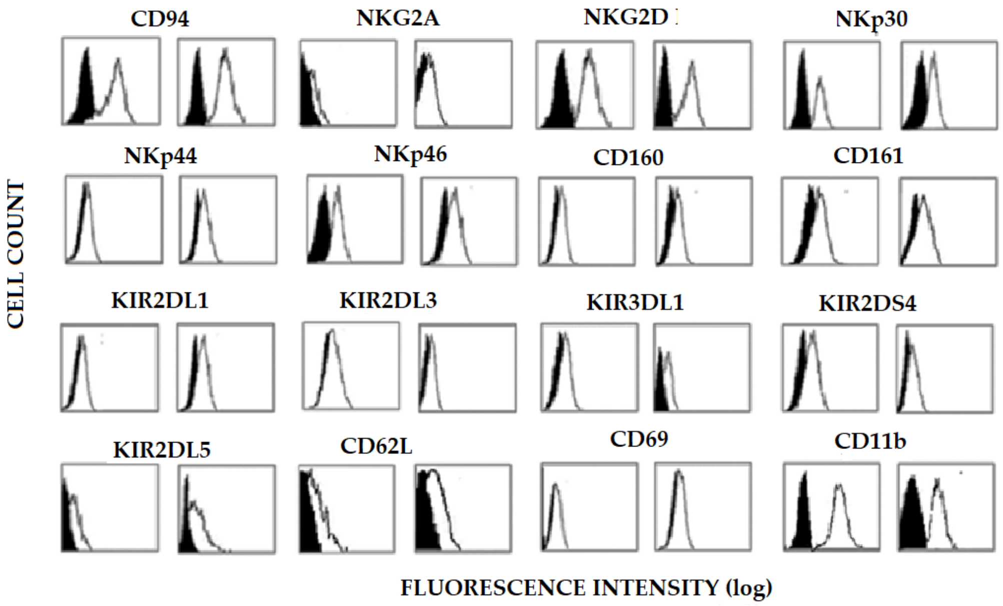

Figure S2. The figure represents the flow cytometry assessment of the different receptors in typical healthy young adults and a healthy elder individual. The histogram in black represents unspecific, irrelevant antibody binding, and the white histogram the positiveness to the antigen analysed. For each antigen, the histogram in the left represents the healthy young adult and in the right, the healthy elder subject.

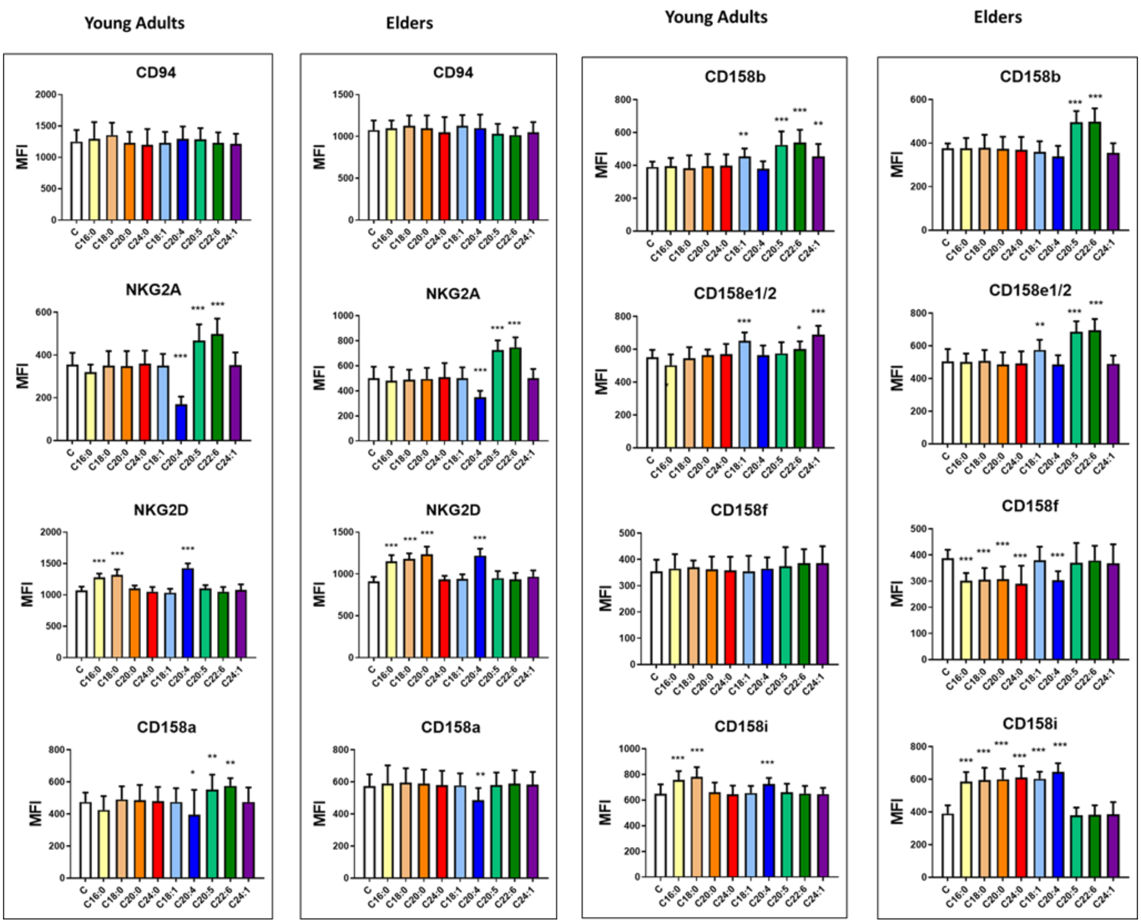

Figure S3. Effect of fatty acids treatment on the mean fluorescence intensity of CD94, NKG2A, NKG2D, CD158a, CD158b, CD158e1/2, CD158f, and CD158i antigens. NK cells purified from the blood of the healthy young adults (23 ± 4 years old) and healthy elders (63 ± 5 years old) were treated with 10 µg/mL concentration of the following fatty acids for 24 h. The expression of the receptors was assessed by flow cytometry. The results represent the percentage mean and SD for each group (*n* = 30). The treatments were compared by one way ANOVA. Bonferroni significance post-test are represented. * $p < 0.01$, ** $p < 0.001$ and *** $p < 0.0001$. No significant differences are recorded for CD94.

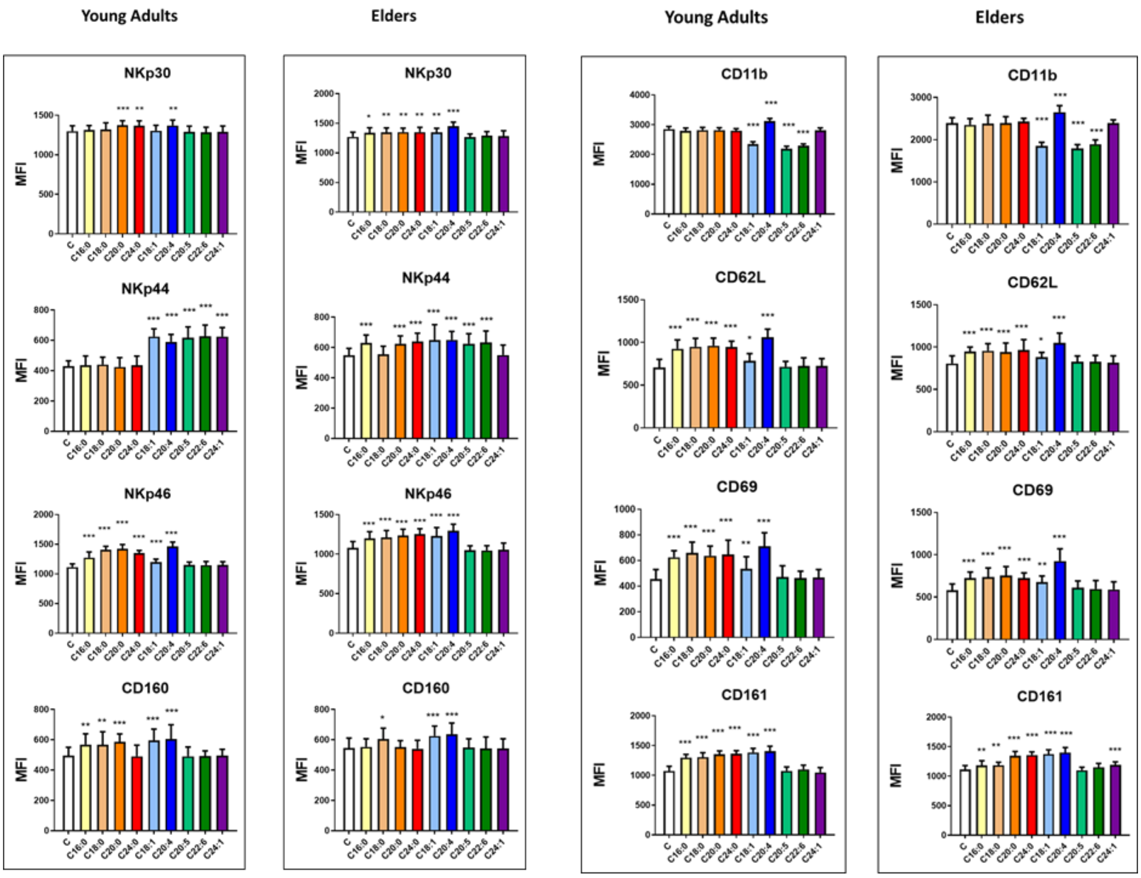

Figure S4. Effect of fatty acids treatment on the mean fluorescence intensity of NKp30, NKp44, NKp46, CD160, CD11b, CD62L, CD69, and CD161 antigens. NK cells purified from the blood of the healthy young adults (23 ± 4 years old) and healthy elders (63 ± 5 years old) were treated with 10 µg/mL concentration of the following fatty acids for 24 h. The expression of the receptors was assessed by flow cytometry. The results represent the percentage mean and SD for each group (n = 30). The treatments were compared by one way ANOVA. Bonferroni significance post-test are represented. * $p < 0.01$, ** $p < 0.001$ and *** $p < 0.0001$.

Table S1. Number of peripheral blood NK cells mm^3 and the% purity after separation.

	Healthy Young Adult Group			Healthy Elderly Group		
	**Lymphocytes**	**NK**	**% Purity**	**Lymphocytes**	**NK**	**% Purity**
1	2438	263	95	2112	203	91
2	2259	237	94	2234	237	95
3	2888	260	93	2427	286	98
4	2205	194	94	2393	258	96
5	3189	322	97	2090	253	97
6	2306	268	96	2699	286	96
7	3123	300	95	2764	271	96
8	2798	285	94	2835	269	95
9	2034	201	91	2243	197	94
10	2238	186	97	2009	207	93
11	2672	283	96	2529	250	98
12	2904	334	92	2454	265	96
13	2151	245	95	2076	199	97
14	2466	266	97	2480	260	95
15	2680	308	95	2577	219	94
16	2893	292	98	3003	228	96

17	2912	285	94	3071	261	91
18	2784	270	96	2362	163	96
19	2655	252	93	2612	196	92
20	2309	203	98	2792	302	90
21	2396	230	90	1917	217	96
22	2478	263	93	2065	207	97
23	3120	337	94	1909	168	95
24	2842	230	95	2790	268	96
25	3161	272	94	2521	232	93
26	2964	287	90	2576	265	98
27	2839	278	90	2357	207	95
28	2871	290	94	2803	241	92
29	2795	296	95	2647	204	93
30	2722	302	93	2530	200	96
Mean ± SD	2669.6 ± 325.6	268.1 ± 39.2	94.3 ± 2.2	2462.6 ± 315.1	234.0 ± 36.0	94.9 ± 2.2
t-Test				**0.02**	**0.001**	**0.3**

Table S2. NK cell markers modulation by fatty acid treatment in cells isolated from the blood of healthy young adults and healthy elder subjects. The table summarises the increase. in green. and the decrease. in red. of expression of NK cell markers under treatment with saturated and unsaturated fatty acids.

Young Adults	SATURATED FATTY ACIDS				UNSATURATED FATTY ACIDS				
					Ω9	Ω6	Ω3	Ω3	Ω9
marker	C16:0	C18:0	C20:0	C24:0	C18:1	C20:4	C20:5	C22:6	C24:1
CD94									
NKG2A						↓	↑	↑	
NKG2D	↑	↑				↑			
CD158a						↓	↑	↑	
CD158b					↑		↑	↑	↑
CD158e1/e2	↓				↑			↑	↑
CD158f									
CD158i	↑	↑				↑			
NKp30			↑	↑		↑			
NKp44					↑	↑	↑	↑	↑
NKp46	↑	↑	↑			↑			

CD160	↑	↑	↑		↑	↑			
CD11b					↓	↑	↓	↓	
CD62L	↑	↑	↑	↑	↑	↑			
CD69	↑	↑	↑	↑	↑	↑			
CD161	↑	↑	↑	↑	↑	↑			

Elders	SATURATED FATTY ACIDS				UNSATURATED FATTY ACIDS				
					Ω9	Ω6	Ω3	Ω3	Ω9
marker	C16:0	C18:0	C20:0	C24:0	C18:1	C20:4	C20:5	C22:6	C24:1
CD94									
NKG2A						↓	↑	↑	
NKG2D	↑	↑	↑			↑			
CD158a						↓			
CD158b							↑	↑	
CD158e1/e2					↑		↑	↑	
CD158f		↓	↓	↓		↓			
CD158i	↑	↑	↑	↑	↑	↑			
NKp30	↑	↑	↑	↑	↑	↑			
NKp44	↑		↑	↑	↑	↑	↑	↑	
NKp46	↑	↑	↑	↑	↑	↑			
CD160		↑			↑	↑			
CD11b		↑	↑	↑	↓	↑	↓	↓	
CD62L	↑	↑	↑	↑	↑	↑			
CD69	↑	↑	↑	↑	↑	↑			

CD161	↑	↑	↑	↑	↑	↑			

Table S3. effect of different concentrations of fatty acid on CD94 expression and cell viability.

**Fatty acid μM**	**Healthy Young Adult Group**		**Healthy Elderly Group**	
	**CD94%**	**Viability%**	**CD94%**	**Viability%**
0	93.6 ± 6.1	98.1 ± 1.3	90.2 ± 8.3	96.3 ± 1.4
1	95.2 ± 3.6	96.6 ± 2.9	88.9 ± 5.2	95.8 ± 3.0
5	92.7 ± 4.5	95.6 ± 3.2	90.6 ± 5.5	97.8 ± 1.6
10	95.5 ± 4.6	96.4 ± 2.5	89.6 ± 9.5	95.3 ± 4.3
20	92.6 ± 6.7	94.8 ± 4.3	92.4 ± 7.1	93.3 ± 5.2
50	92.6 ± 5.4	83.9 ± 8.3	83.6 ± 9.5	72.6 ± 12.1

The values represent the mean and SD of the values recorded for all the fatty analysed in a group of 5 individuals of each group. The value of 10 μM was chosen to be optimal for cell viability. CD94 expression and the equivalent amount of each fatty acid detected in the plasma of the volunteers.
